# Supplementary material for: Regulating electrostatic phenomena by cationic polymer binder for scalable high-areal-capacity Li battery electrodes
Source: Nat Commun. 2023 Sep 15;14:5721. doi: 10.1038/s41467-023-41513-1 (PMC10504278; doi:10.1038/s41467-023-41513-1)
Supplement: Supplementary file 1 — Supplementary Information File [file 41467_2023_41513_MOESM1_ESM.pdf]

Supplementary Information

Regulating electrostatic phenomena by cationic polymer binder for scalable high-areal-capacity Li battery electrodes

Jung-Hui Kim[1,5], Kyung Min Lee[2,5], Ji Won Kim[3], Seong Hyeon Kweon[2], Hyun-Seok Moon[1], Taeeun Yim[3✉], Sang Kyu Kwak[4✉], Sang-Young Lee[1✉]

[1]Department of Chemical and Biomolecular Engineering, Yonsei University, Seoul 03722, Republic of Korea

[2]Department of Energy Engineering, School of Energy and Chemical Engineering, Ulsan National Institute of Science and Technology (UNIST), Ulsan 44919, Republic of Korea

[3]Department of Chemistry, Incheon National University, Incheon 22012, Republic of Korea

[4]Department of Chemical and Biological Engineering, Korea University, 145 Anam-ro, Seongbuk-gu, Seoul 02841, Republic of Korea

[5]These authors contributed equally to this work: Jung-Hui Kim, Kyung Min Lee

[✉]email: yte0102@inu.ac.kr; skkwak@korea.ac.kr; syleek@yonsei.ac.kr

Supplementary Figures

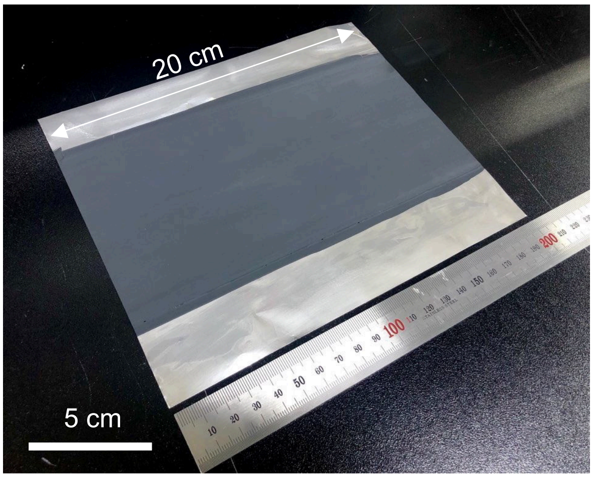

Supplementary Fig. 1 | Photograph of the scalable cationic semi-interpenetrating polymer network (c-IPN) cathode (*C*/*A* = 18 mAh cm^{-2}) adopted for a double-stacked pouch-type full cell (area = 34 × 34 (mm × mm)), which was prepared using the commercial slurry-cast electrode fabrication method.

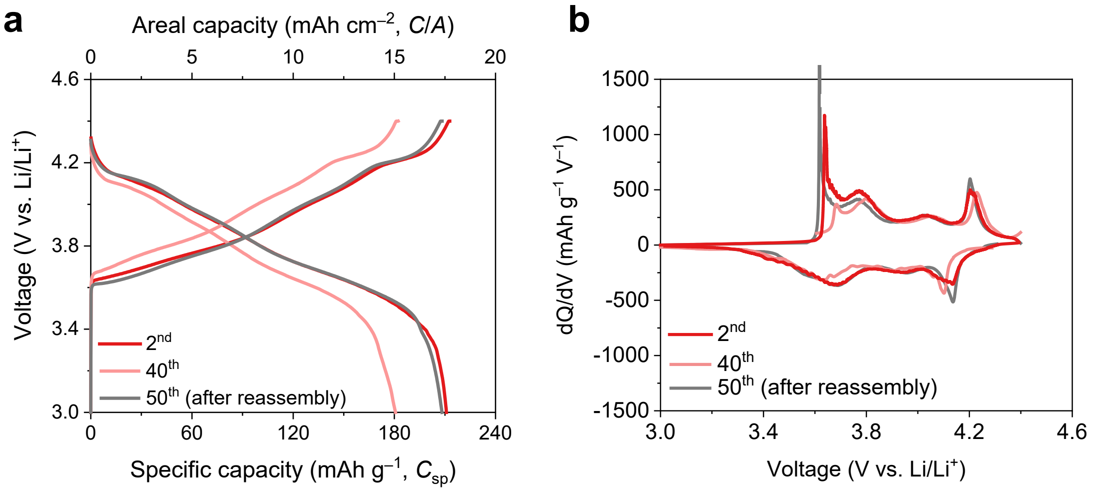

Supplementary Fig. 2 | a,**b**, Galvanostatic charge/discharge profiles (**a**) and corresponding dQ/dV plots (**b**) of the double-stacked pouch cell at 2nd, 40th, and 50th (after reassembly) cycles, in which the cell was cycled at charge/discharge current density of 0.9 mA cm^{-2}/1.8 mA cm^{-2} and voltage range of 3.0–4.4 V. The cycled cathodes were reassembled with fresh Li-metal anode, separator, and liquid electrolyte at 50th cycle.

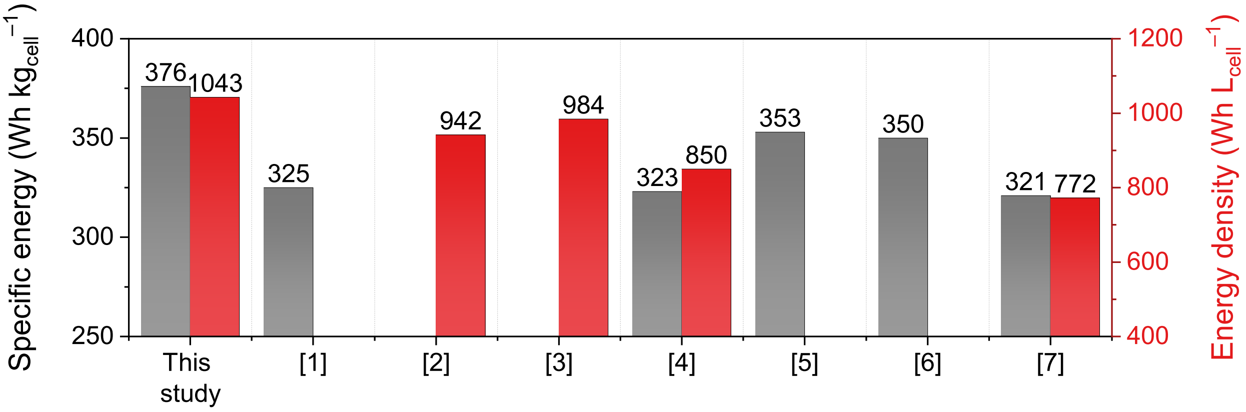

Supplementary Fig. 3 | Comparison of specific energies and energy densities of the high-energy-density pouch cells fabricated in this study and previously reported high-energy-density pouch cells[1-7]. The specific energies and energy densities were estimated based on the total mass and volume (including packaging substances) of the pouch cells. The cell parameters are described in **Supplementary Table 3**.

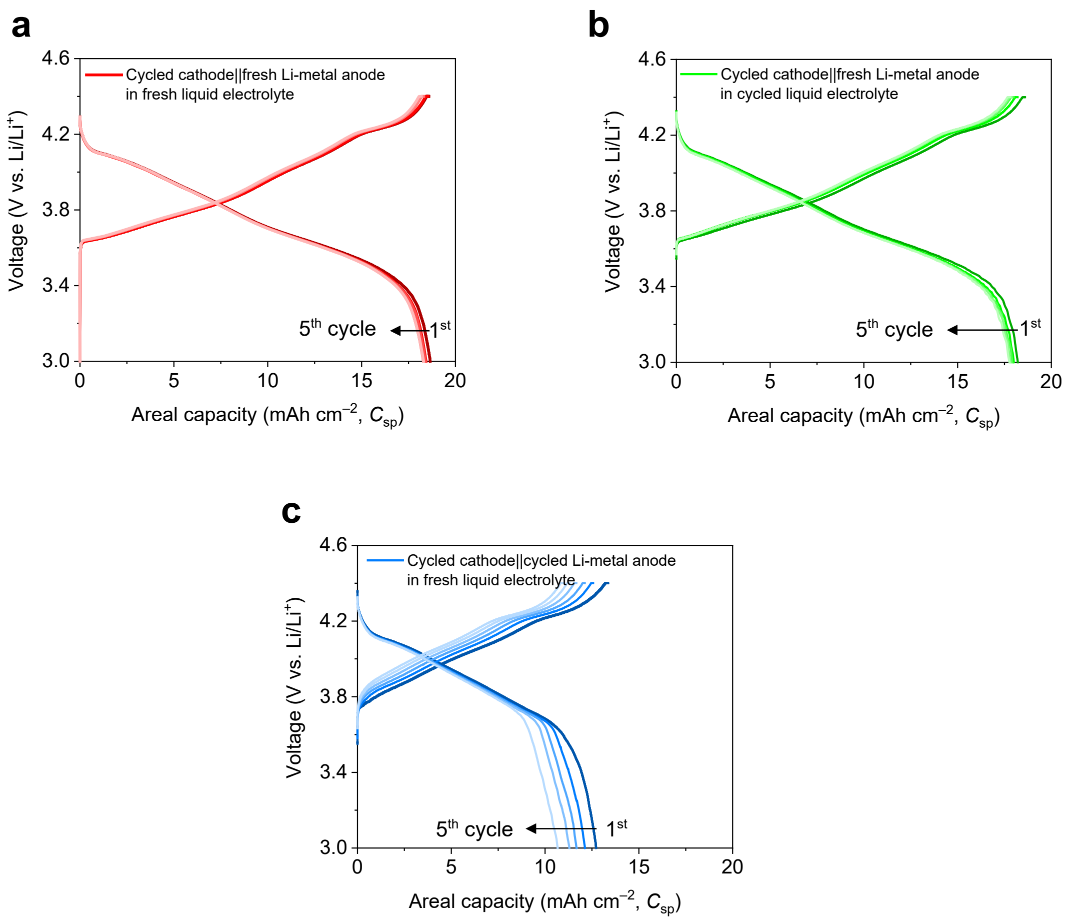

Supplementary Fig. 4 | a-c. Galvanostatic charge/discharge profiles of the reassembled pouch cells: A model cell #1 (cycled cathode||fresh Li-metal anode in fresh liquid electrolyte) (**a**), A model cell #2 (cycled cathode||fresh Li-metal anode in cycled liquid electrolyte) (**b**), and a model cell #3 (cycled cathode||cycled Li-metal anode in fresh liquid electrolyte) (**c**). The cells were cycled at charge/discharge current rate of 0.05 C/0.1 C (= 0.9 mA cm^{-2}/1.8 mA cm^{-2}) and voltage range of 3.0 – 4.4 V.

To identify a major cause of the cycling fading of the Li-metal full cells shown in **Fig. 2d**, three model cells were prepared as follows: model cell #1 (cycled cathode||fresh Li-metal anode in fresh liquid electrolyte) vs. model cell #2 (cycled cathode||fresh Li-metal anode in cycled liquid electrolyte) vs. model cell #3 (cycled cathode||cycled Li-metal anode in fresh liquid electrolyte). The model cell #1 and #2 returned to an initial voltage profile together with the high-*C*/*A* value (~ 18 mAh cm^{-2}). In

comparison, the model cell #3 failed to return to the initial voltage profile even in the presence of a fresh liquid electrolyte and showed rapid capacity fading with cycling. This result exhibits that the cycled Li-metal anode is a major cause of the cycling degradation of the full cells with the high-*C*/*A* cathodes.

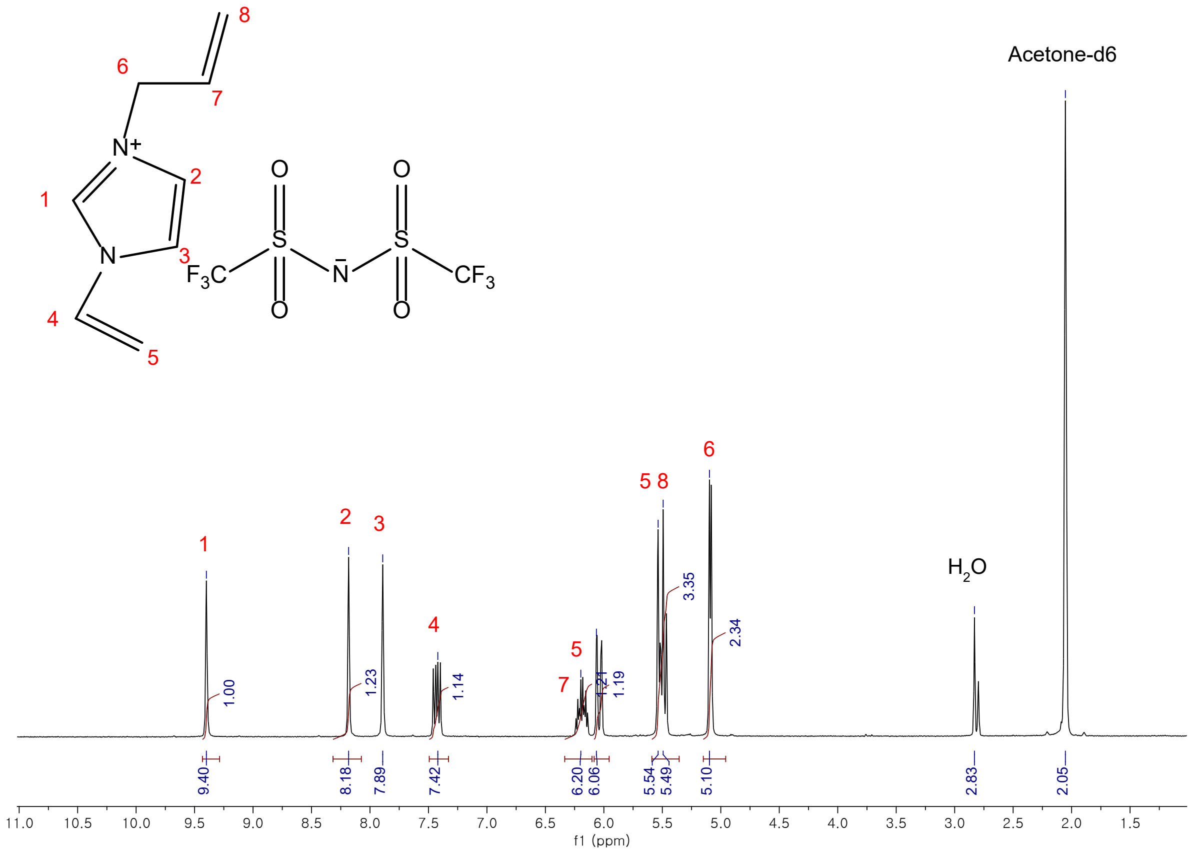

Supplementary Fig. 5 | ^{1}H nuclear magnetic resonance (NMR) spectra and chemical structure of the synthesized 1-vinyl-3-allylimidazolium bis(trifluoromethanesulfonyl)imide (VAI-TFSI).

The VAI-TFSI was synthesized through the bromination of 1-vinylimidazole followed by anion exchange with TFSI$^-$ (details on the synthetic procedure are described in the Methods section).

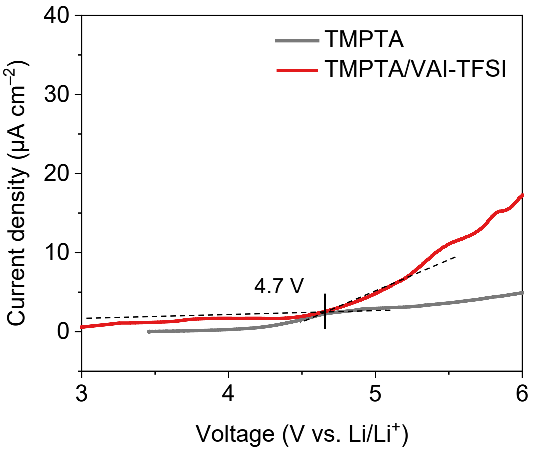

Supplementary Fig. 6 | Electrochemical stability window (measured by the linear sweep voltammetry (LSV)) of the crosslinked trimethylolpropane triacrylate (TMPTA) and TMPTA/VAI-TFSI.

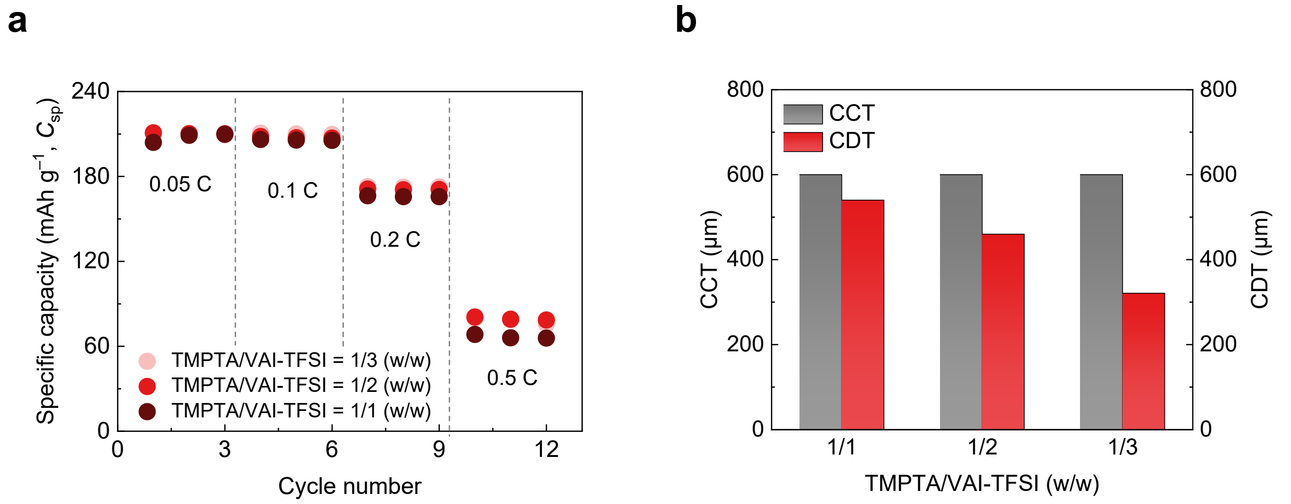

Supplementary Fig. 7 | a, Discharge rate capabilities of the electrodes (M/A = 65 mg cm^{-2}) as a function of the TMPTA/VAI-TFSI composition ratio. **b**, Critical cracking thickness (CCT) and critical delamination thickness (CDT) of the electrodes (M/A = 65 mg cm^{-2}) as a function of the TMPTA/VAI-TFSI composition ratio. CCT represents the critical electrode thickness upon the crack generation during electrode drying. CDT represents the critical electrode thickness upon the delamination of the electrode layer from the Al current collector during roll-pressing.

With increasing the VAI-TFSI content, the discharge rate capability of the electrodes tended to increase. However, the adhesion strength of the electrodes was deteriorated due to the poor binding capability of the VAI-TFSI. From these results, the optimal composition ratio of TMPTA/VAI-TFSI was determined to be 1/2 (w/w).

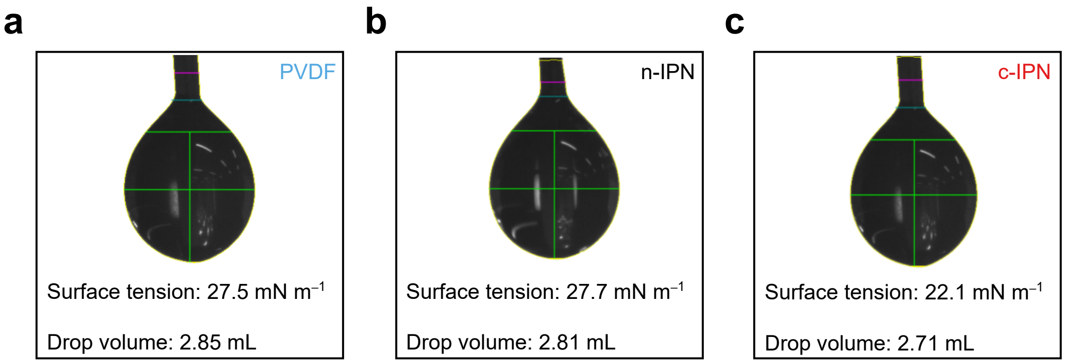

Supplementary Fig. 8 | a-c, Photographs of the pendant drop tensiometry used to measure the surface tension of polyvinylidene fluoride (PVDF) (**a**), n-IPN (**b**), and c-IPN (**c**) electrode slurries.

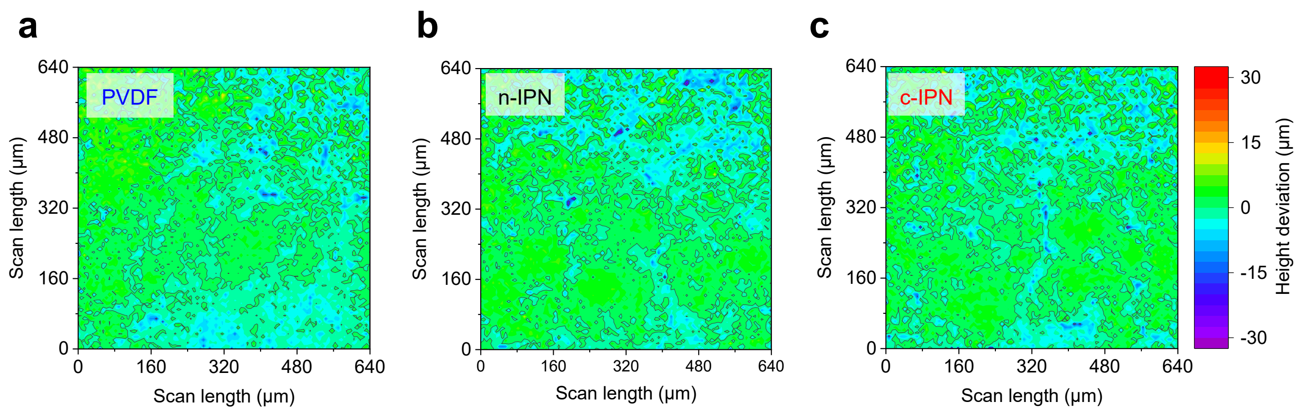

Supplementary Fig. 9 | a-c, Laser scanning confocal microscopy (LSCM) surface topography images of PVDF (**a**), n-IPN (**b**), and c-IPN (**c**) electrodes with a low-*M*/*A* (= 26 mg cm^{-2}).

The height deviation could cause the electrodes to experience uneven distribution of pressure during roll-pressing steps, resulting in dimensional instability and nonuniformity of the resultant electrodes.

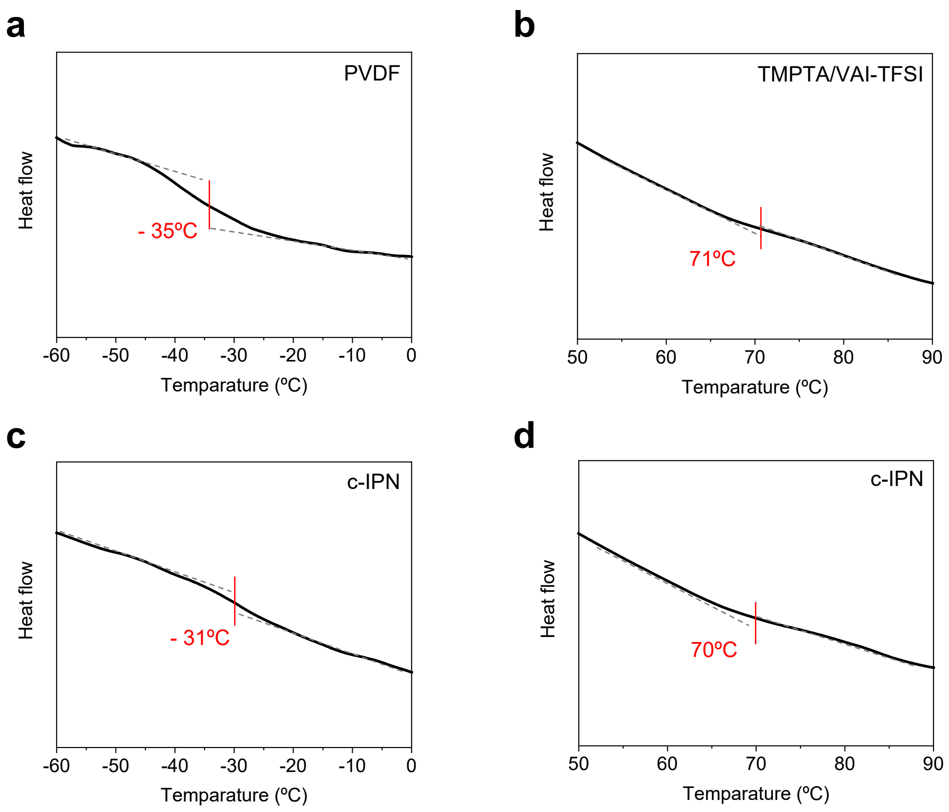

Supplementary Fig. 10 | a-d. Differential scanning calorimetry (DSC) profiles showing the glass transition temperatures (T_g) of the pristine PVDF film (**a**), pristine TMPTA/VAI-TFSI crosslinked copolymer film (**b**), and c-IPN film (**c**,**d**).

The c-IPN film showed two different glass transition temperatures (- 31℃ and 70℃), both of which were almost matched with to those of pristine PVDF (-35℃) and crosslinked copolymer (71℃), respectively. This result indicates the phase separation of the PVDF and crosslinked copolymer in the c-IPN, which is a typical feature of IPN structure.

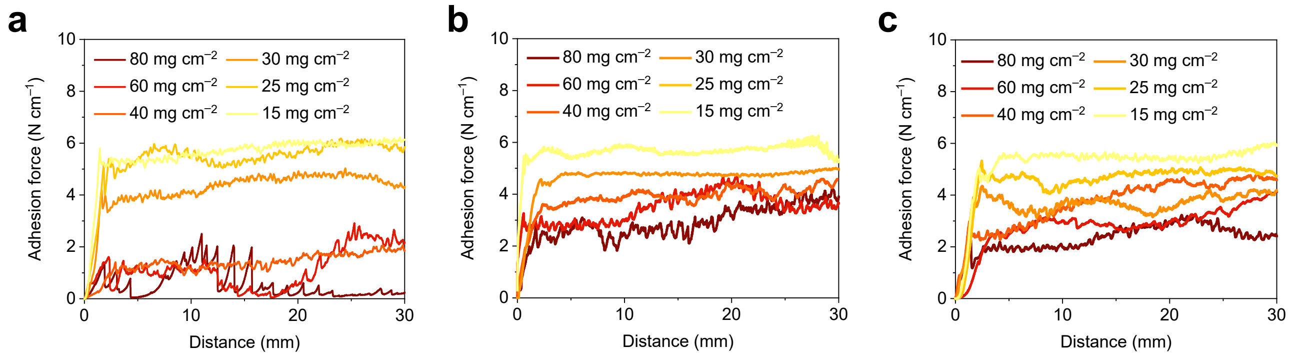

Supplementary Fig. 11 | a-c, Adhesion forces between electrode active layers and Al current collectors (measured by 180° peel-off test) of the PVDF (**a**), n-IPN (**b**), c-IPN (**c**) electrodes with various *M*/*A* values.

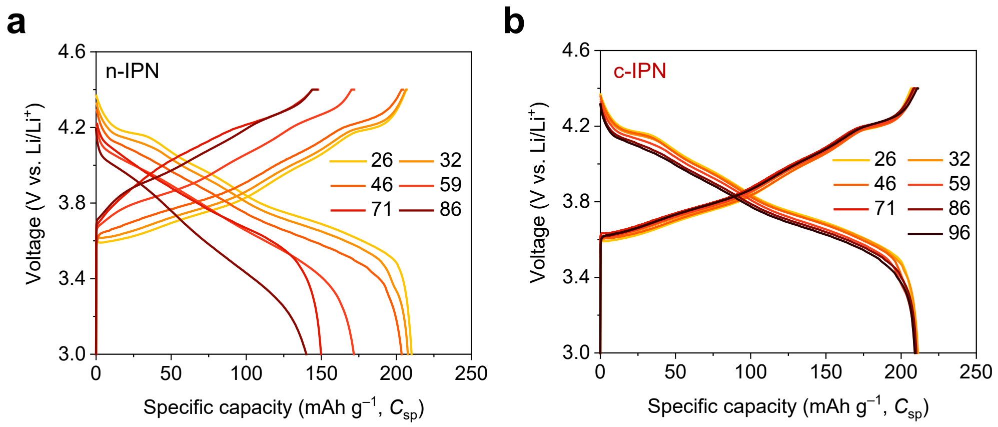

Supplementary Fig. 12 | a,**b**, Galvanostatic charge/discharge profiles of the n-IPN (**a**) and c-IPN (**b**) cathodes as a function of their *M*/*A* (= 26–96 mg cm^{-2}) at a charge/discharge current rate of 0.05 C/0.1 C and voltage range of 3.0–4.4 V, in which the cathodes were coupled with 100 μm Li-metal anodes (*C*/*A* = 20 mAh cm^{-2}).

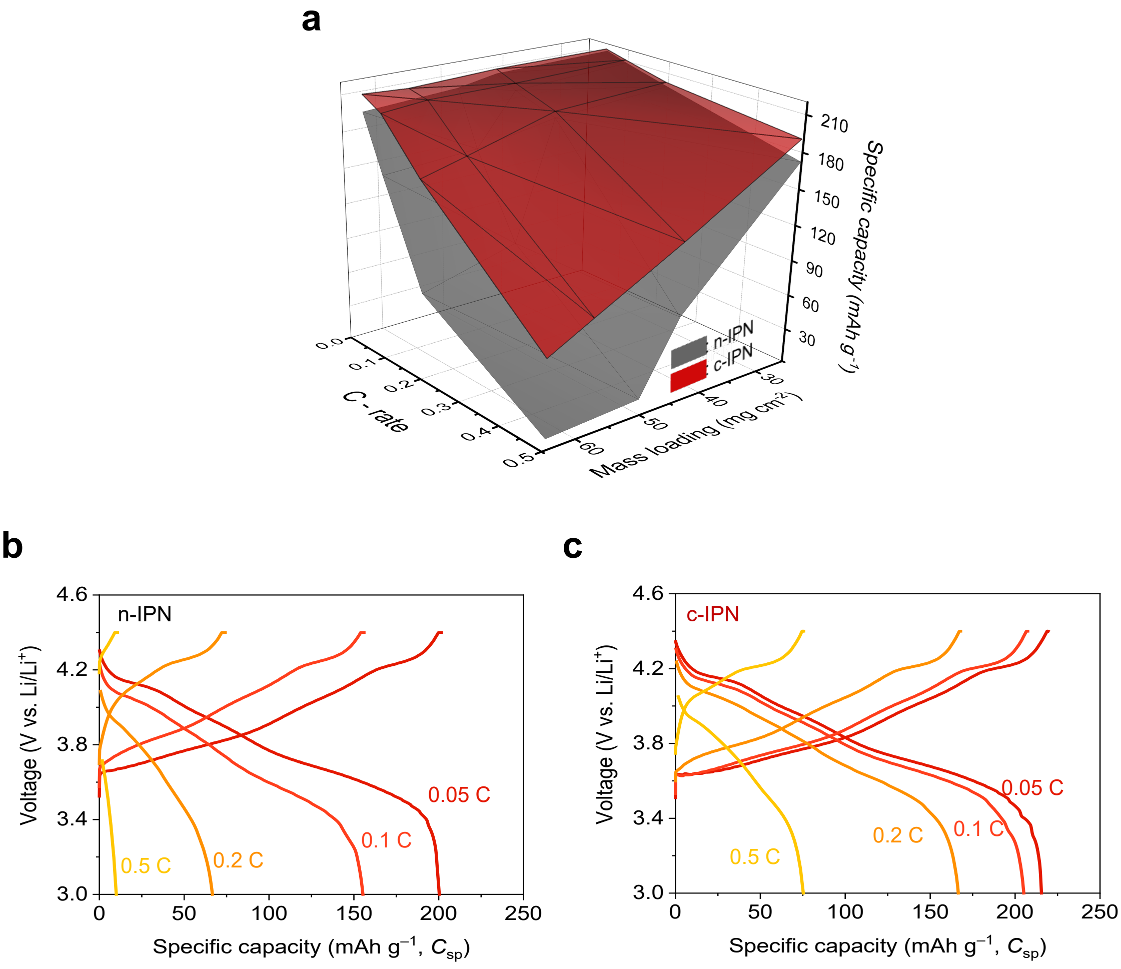

Supplementary Fig. 13 | a, Discharge rate capability (expressed as C_{sp}) of the c-IPN (vs. n-IPN) cathodes coupled with 100 μm Li-metal anodes (C/A = 20 mAh cm^{-2}) as a function of their M/A. **b**,**c**, Galvanostatic charge/discharge profiles of the cells with n-IPN (**b**), and c-IPN (**c**) cathodes with high-M/A (= 65 mg cm^{-2}) under varying discharge current rates of 0.05 C (= 0.68 mA cm^{-2})–0.5 C (= 6.75 mA cm^{-2}).

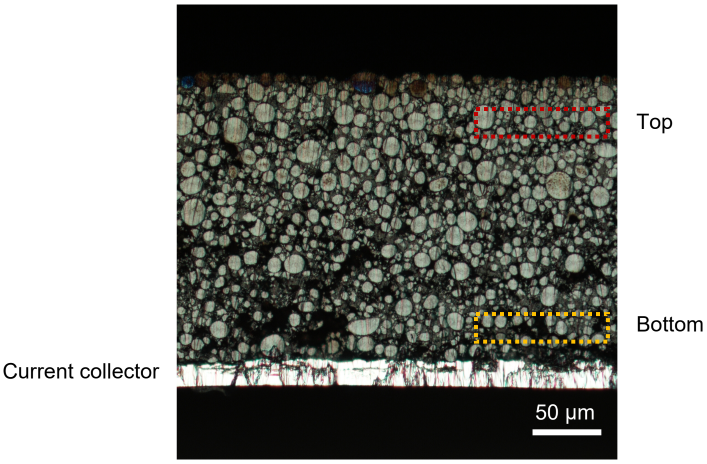

Supplementary Fig. 14 | Cross-sectional optical micrograph of the control n-IPN electrode (M/A = 65 mg cm^{-2}).

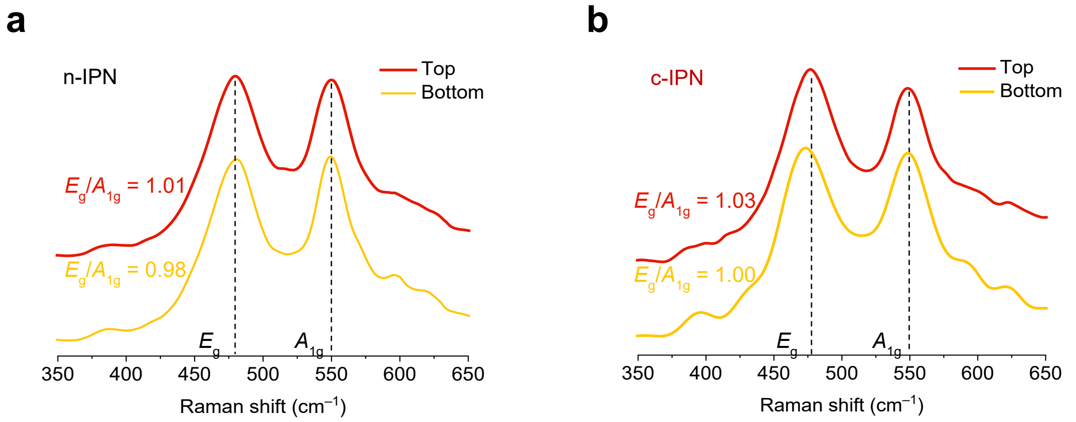

Supplementary Fig. 15 | a,b, Raman spectra and intensity ratio of E_g/A_{1g} of the n-IPN (**a**) and c-IPN (**b**) cathodes with a low-*M*/*A* of 26 mg cm^{-2} at the top and bottom regions after being charged at a current rate of 0.2 C (= 1.1 mA cm^{-2}), in which E_g and A_{1g} represent the bending mode of metal-oxygen-metal in *a*/*b*-axis direction and stretching mode of metal-oxygen complex in *c*-axis direction, respectively.

In comparison to the results of high-*M*/*A* cathodes, no significant difference in the E_g/A_{1g} was observed at the low-*M*/*A* cathodes.

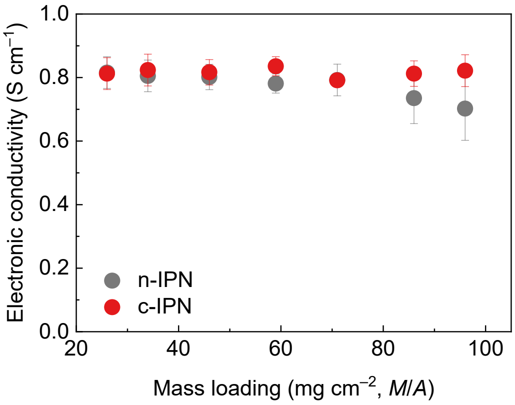

Supplementary Fig. 16 | Comparison of the electronic conductivity (measured using a four-point probe station) between the c-IPN and control n-IPN cathodes as a function of the *M*/*A*. The electronic conductivities of the cathodes were negligibly different at a *M*/*A* of 65 mg cm^{-2}.

This result indicates that the difference in the E_g/A_{1g} intensity ratios between the two cathodes is mainly attributed to a sluggish ion migration in the through-thickness direction of the n-IPN cathode, which is consistent with previous studies on high-*M*/*A* electrodes that reported the importance of ion transport in achieving the redox uniformity[8,9].

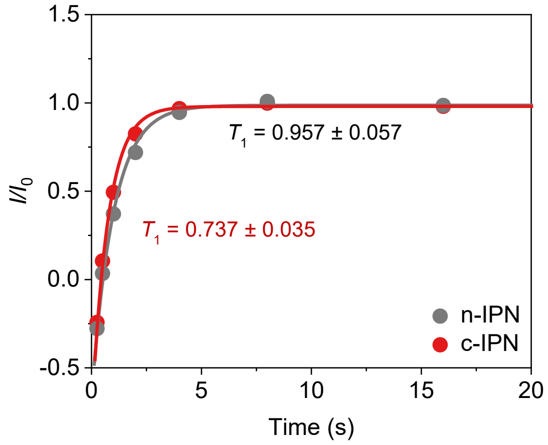

Supplementary Fig. 17 | Inversion-recovery plots obtained from ^{7}Li MAS NMR spectra. The spin–lattice relaxation time (T_1) was calculated using the following equation (1)[10]:

(1) $$I = I_0(1 - exp^{-t/T_1})$$

Where I is the peak intensity at time t, I_0 is the saturation intensity, respectively.

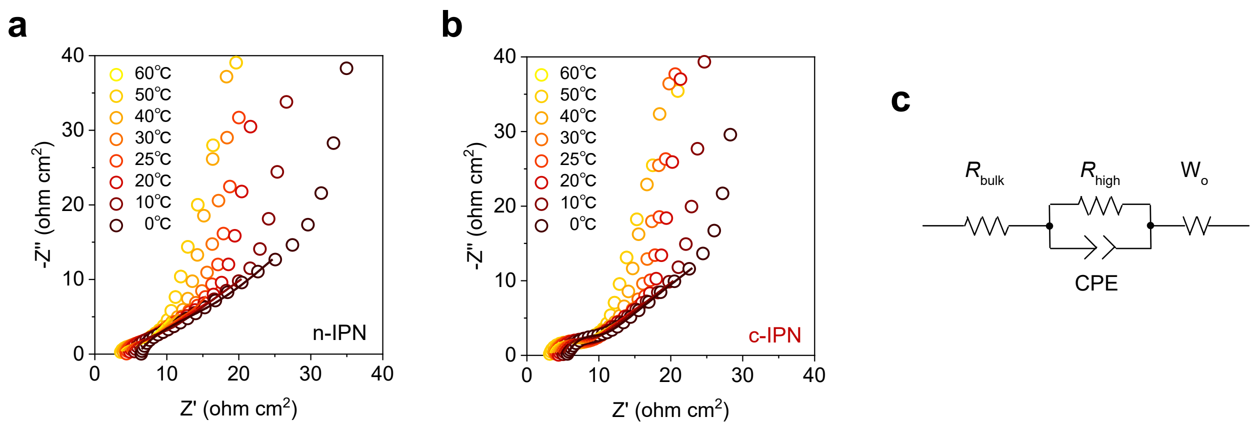

Supplementary Fig. 18 | a,b, Nyquist plots of the symmetric cell (electrode||electrode) with high-*M*/*A* (= 65 mg cm^{-2}) n-IPN (**a**), and c-IPN (**b**) cathodes at a fully lithiated state, in which symbols and solid lines represent experimental data and fitted curves based on a transmission line equivalent circuit model (TLM), respectively. **c**, Equivalent circuits using the generalized finite length Warburg element open circuit terminus (W_o).

A slope observed in the low frequency region of Nyquist plots is known to reflect ionic resistance inside electrodes ($R_{ion}/3$)[11]. The obtained R_{ion} was used to calculate the ion conductivity ($\sigma_{electrode} = 1/R_{ion}$) inside the cathode. W_o was used to estimate $R_{ion}/3$[12].

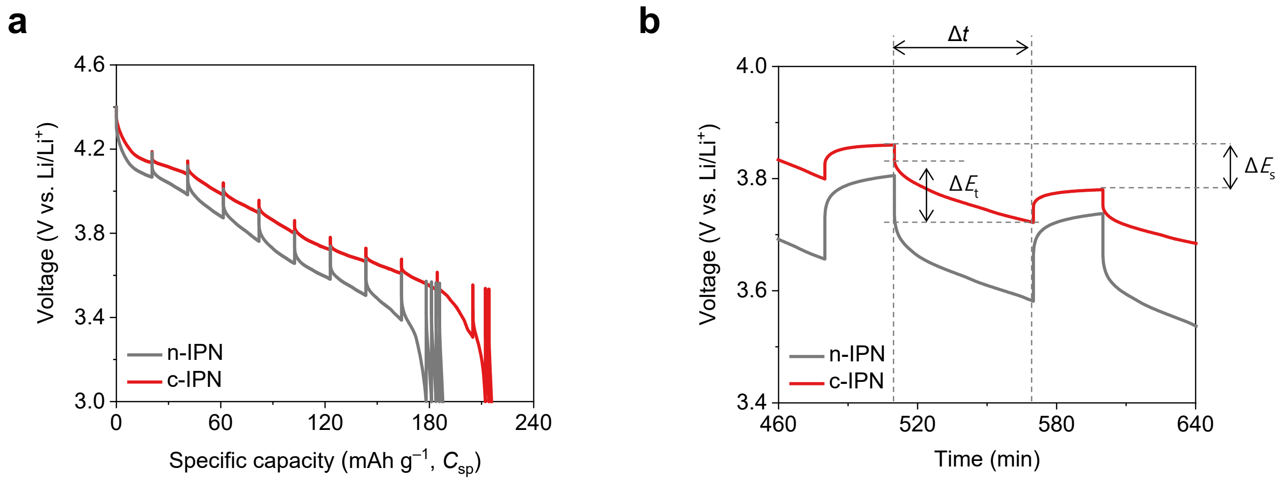

Supplementary Fig. 19 | a, Galvanostatic intermittent titration technique (GITT) profiles of the high-*M*/*A* (= 65 mg cm^{-2}) cathodes (c-IPN vs. n-IPN) upon the repeated current stimuli at discharge current rate of 0.1 C (= 1.35 mA cm^{-2}). **b**, GITT profiles showing the discharging step of the cells with the cathodes. The Li^+ diffusion coefficients (D_{Li^+}) were calculated using the following equation (2)[13]:

(2) $$D_{Li^+} = \frac{4}{\pi \Delta \tau} \left(\frac{m_B V_M}{M_B S}\right) \left(\frac{\Delta E_S}{\Delta E_t}\right)^2$$

where m_B is assigned to the mass of the electrode active material, S is the geometric area of the electrode, M_B is the molar mass of the electrode material, V_M is the molar volume of the electrode material, and other parameters (Δt, ΔE_t and ΔE_s) in the equation are displayed in the GITT profiles shown above.

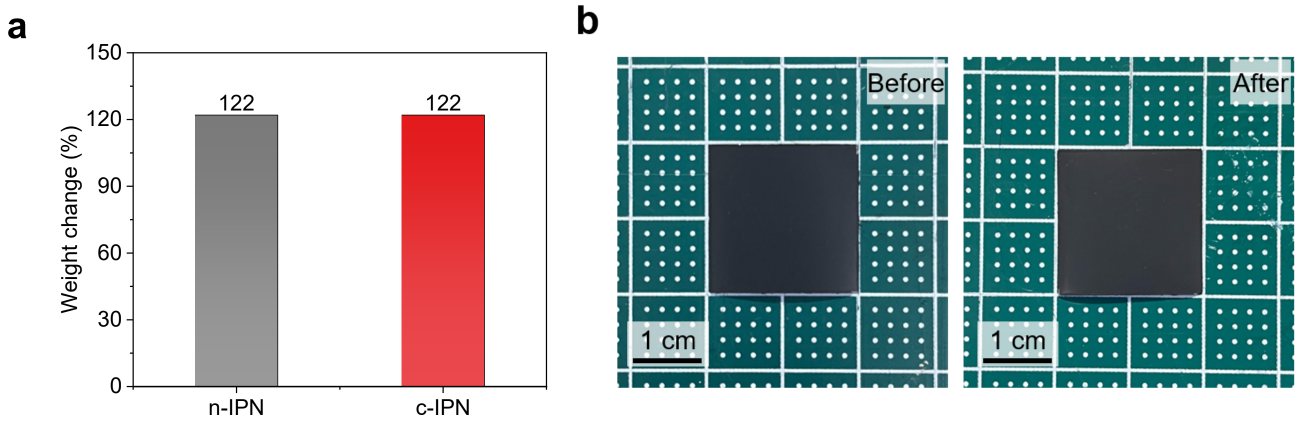

Supplementary Fig. 20 | a, Weight change of the c-IPN (vs. n-IPN) cathodes after being soaked in the liquid electrolyte (1 M $LiPF_6$ in EC/DEC = 1/1 (v/v)) for 2 h. **b**, Photographs of the c-IPN cathode before (left) and after (right) the electrolyte uptake.

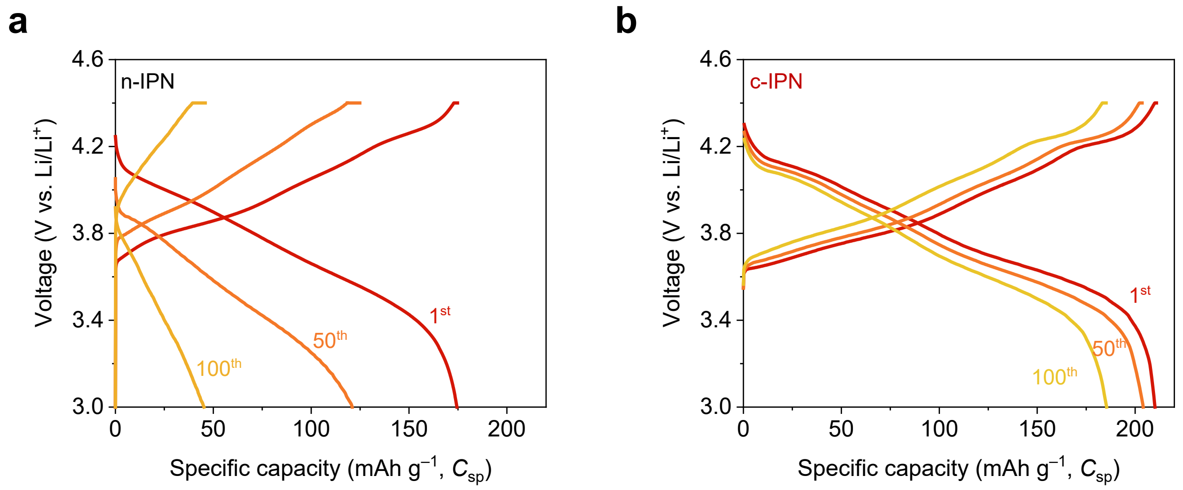

Supplementary Fig. 21 | a,**b**, Galvanostatic charge/discharge profiles of the Li-metal full cells (cathode (C/A = 13.5 mAh cm^{-2})||Li-metal anode (C/A = 20 mAh cm^{-2}, N/P ratio = 1.5) at the 1st, 50th and 100th cycles: n-IPN (**a**) and c-IPN (**b**) cathodes. The cells were cycled at a charge/discharge current density of 0.68 mA cm^{-2}/1.35 mA cm^{-2}and voltage range of 3.0–4.4 V.

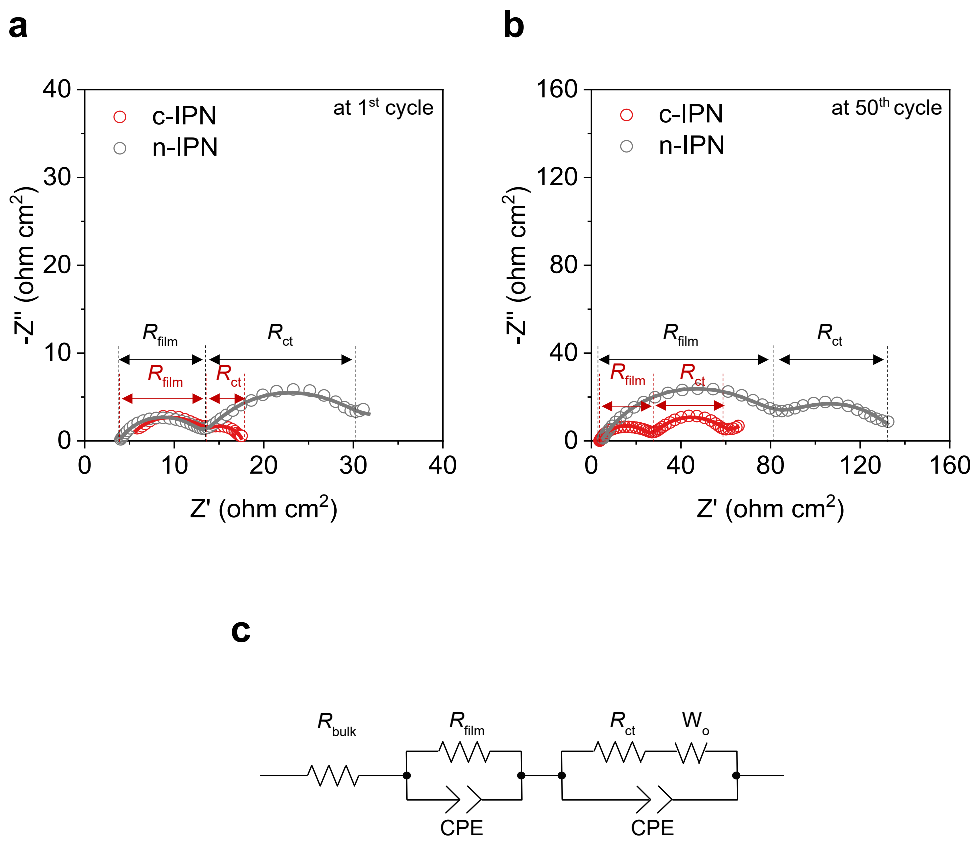

Supplementary Fig. 22 | a,b, Nyquist plots of the Li-metal full cells (n-IPN cathode vs. c-IPN cathode at 100% SOC) at 1st (**a**), and 50th cycle (**b**). The symbols and solid lines represent experimental data and fitted curves, respectively. **c**, Equivalent circuits used to fit the curves[14]. The cathodes with an M/A of 65.0 mg cm^{-2} (corresponding to C/A = 13.5 mAh cm^{-2}) were assembled with Li-metal anodes (C/A = 20.0 mAh cm^{-2}, N/P ratio = 1.5).

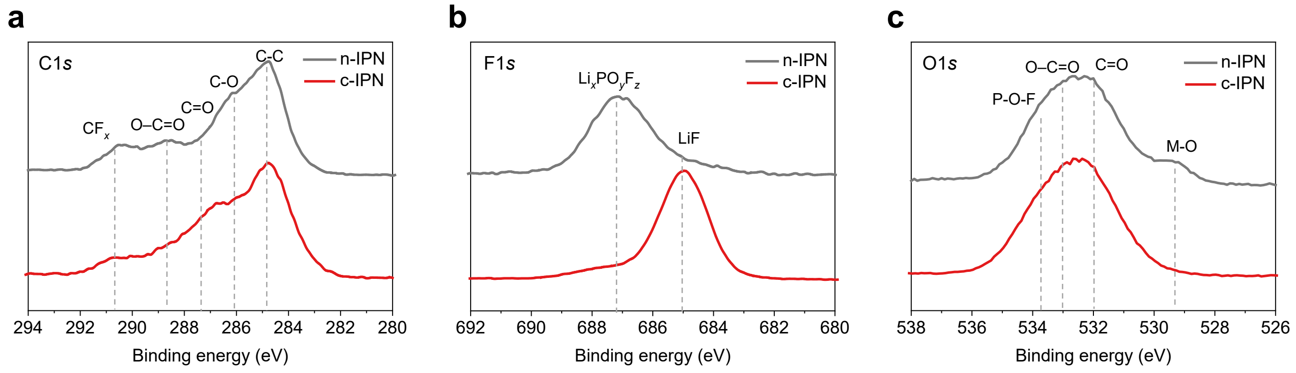

Supplementary Fig. 23 | a-c, X-ray photoelectron spectroscopy (XPS) profiles of the cycled NCM811 in the cathodes (n-IPN vs. c-IPN): C1*s* (**a**), F1*s* (**b**), and O1*s* (**c**).

The XPS F1*s* spectra of the cycled NCM811 in the c-IPN cathode exhibited the predominant presence of LiF (at 685.5 eV). The LiF in the CEI layers is known to beneficially affect cycling stability[15,16]. This result was confirmed by detecting a strong peak at 529.8 eV (assigned to M (metal)-O species)[17], in the O1*s* spectra that accounts for the severe dissolution of transition metal ions in the n-IPN cathode.

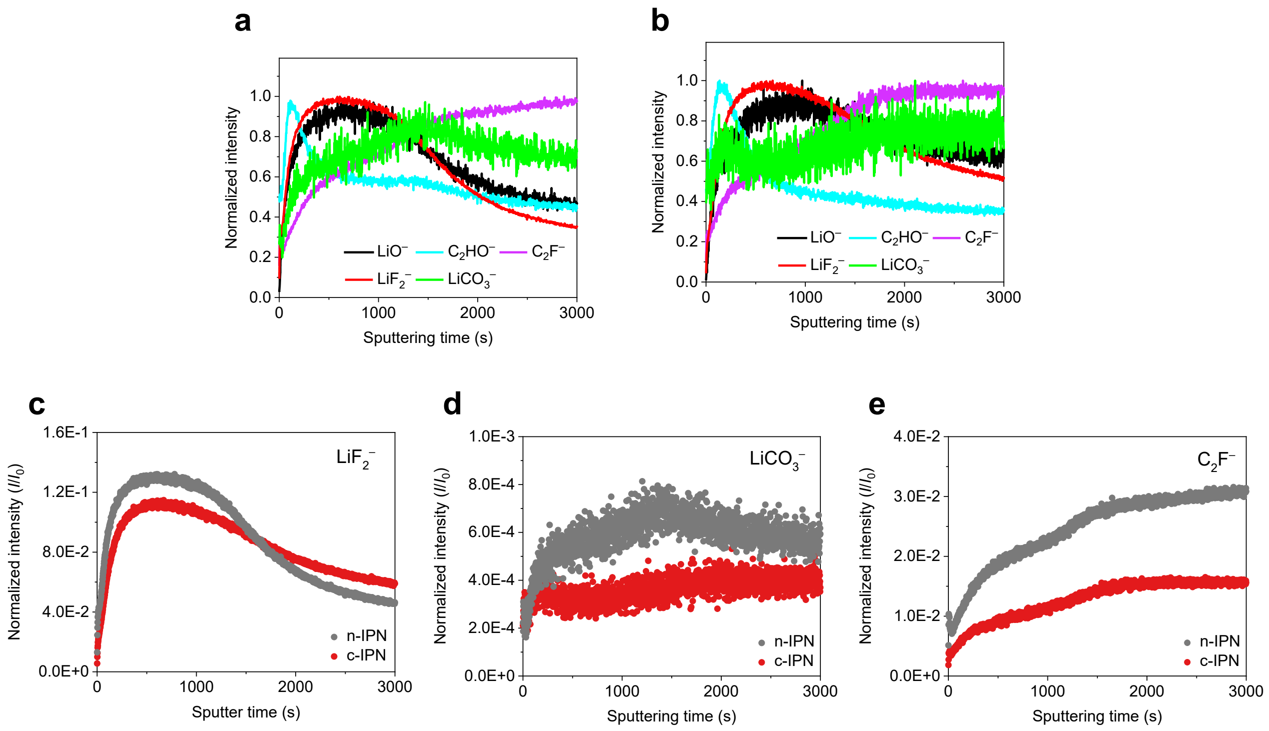

Supplementary Fig. 24 | a,**b**, Normalized time-of-flight secondary ion mass spectrometry (TOF-SIMS) depth profiling of the cycled n-IPN (**a**) and c-IPN (**b**) cathodes. **c**-**e**, Comparison of the TOF-SIMS depth profiles between the cycled n-IPN and c-IPN cathodes: LiF_2^- (**c**), $Li_2CO_3^-$, and C_2F^- (**e**).

In comparison to the control n-IPN cathode, the c-IPN cathode showed the lower intensity of organic components (such as $Li_2CO_3^-$ and C_2F^-) and the higher intensity of inorganic LiF_2^- in the inner layer.

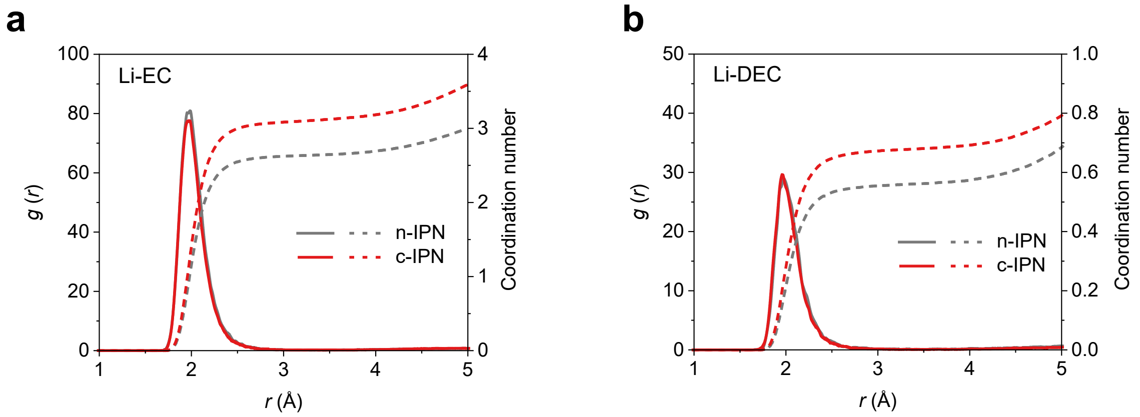

Supplementary Fig. 25 | a,**b**, Radial distribution function (RDF) profiles and coordination number (CN; between Li^+ and solvents in the liquid electrolyte): EC (**a**), and DEC (**b**) at the binder (n-IPN vs. c-IPN)–electrolyte interface. The Li^+ solvation structure of the liquid electrolyte inside the cathodes was quantitively elucidated by analyzing the RDF of Li^+–solvents and calculating the CN.

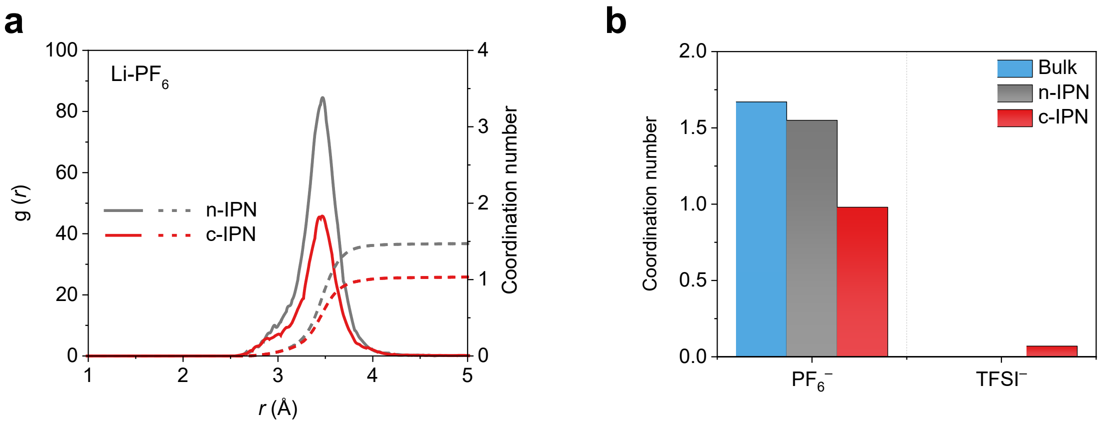

Supplementary Fig. 26 | a,**b**, RDF profiles (**a**) and CN (**b**) (between Li^{+} and PF_6^{-} in the liquid electrolyte) at the binder (n-IPN vs. c-IPN)–electrolyte interface and bulk electrolyte.

The c-IPN binder showed the lower CN compared to the n-IPN binder and the bulk electrolyte, indicating that PF_6^{-} of the liquid electrolyte inside the c-IPN cathode could be less coordinated by solvents because of the electrostatic attraction between PF_6^{-} and c-IPN binder.

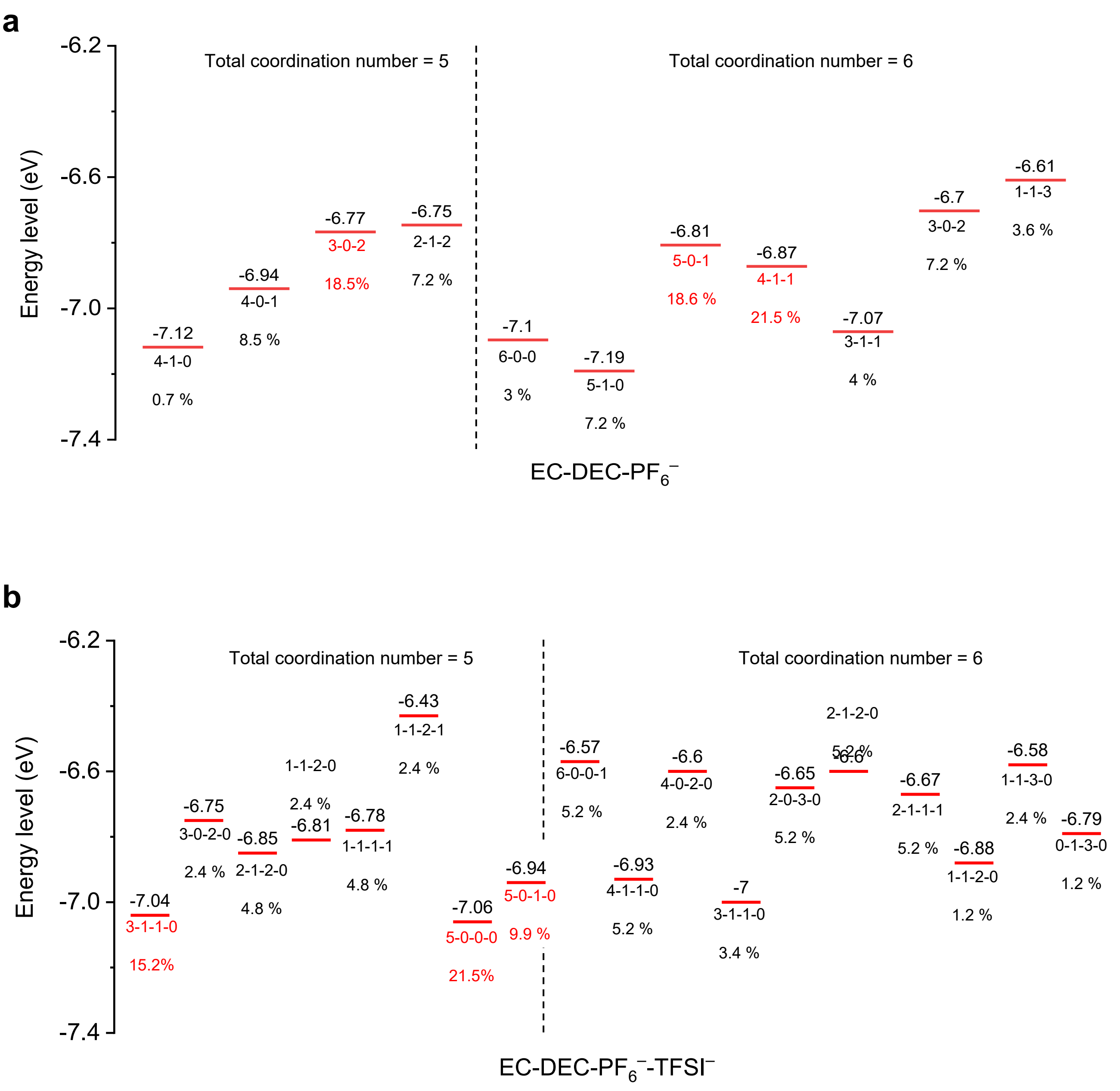

Supplementary Fig. 27 | a,b, Highest occupied molecular orbital (HOMO) energy levels and relative ratios of the Li^+ solvation structures in the liquid electrolyte (1M $LiPF_6$ in EC/DEC = 1/1 (v/v)): n-IPN (**a**) and c-IPN (**b**) cathode. The major Li^+ solvation structures are highlighted in red. The Li^+ solvation structures are denoted as *X-Y-Z-(A)*, where *X* is the number of EC molecules coordinated with Li^+, *Y* is the number of DEC molecules coordinated with Li^+, *Z* is the number of PF_6^- coordinated with Li^+ and *A* is the number of $TFSI^-$ coordinated with Li^+.

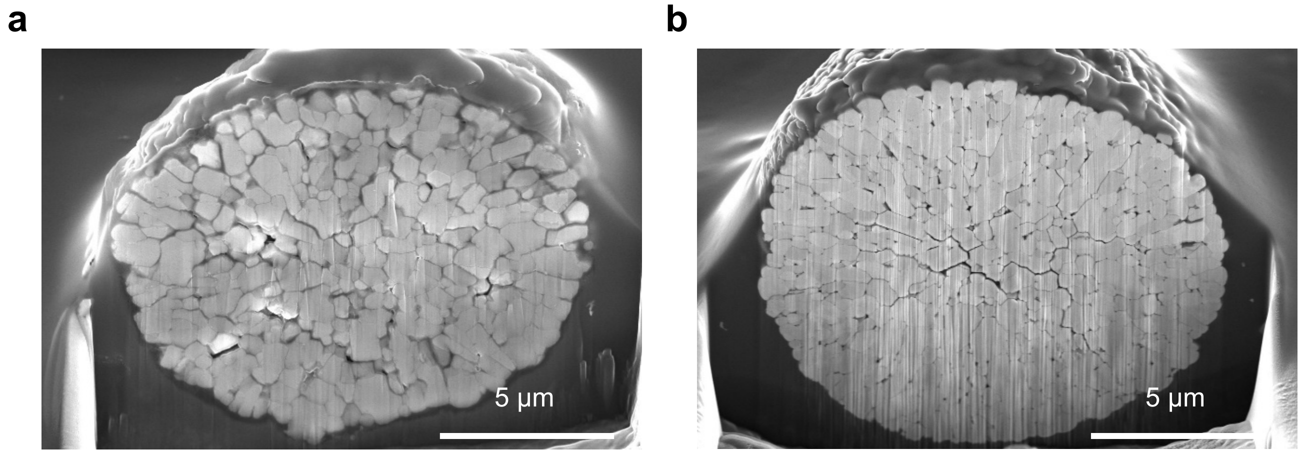

Supplementary Fig. 28 | a,**b**, Cross-sectional scanning electron microscopy (SEM) images of the cycled NCM811 particles in the n-IPN (**a**), and c-IPN (**b**) cathodes.

Into the intergranular cracks, the electrolyte could infiltrate and continuously trigger unwanted interfacial side reactions with fresh primary particles[18,19], thus deteriorating the cycling retention.

Supplementary Tables

Supplementary Table 1 | Details on the calculation of the specific energies and energy densities (excluding packaging substances) of coin cells with c-IPN cathodes.

The specific energy of the cell (shown in **Fig. 2a**) is calculated using the following equation (3)[20],

$$(3)\qquad \text{Specific energy (Wh kg}^{-1}) = \frac{\text{Energy}}{\text{Mass of cell}} = \frac{\text{Nominal volatge} \times C}{M_{cathode} + M_{anode} + M_{separator} + M_{electrolyte}}$$

where the M_{cathode}, M_{anode}, $M_{\text{separator}}$ and $M_{\text{electrolyte}}$ are the mass of cathode (including Al current collector), anode (including a Cu current collector), separator and injected electrolyte. *C* indicates the discharge capacity. The cells were composed of cathodes with various *C*/*A* values and 100 μm Li-metal (corresponding to 20 mAh cm^{-2}). The electrolyte mass/cell capacity (*E*/*C*) ratio in the cell was set as 2.5 g Ah^{-1}. The nominal voltage of the cell was estimated based on the integral of discharge profile divided by the discharge capacity.

C/*A* (mAh cm^{-2})	*C* (mAh)	M_{cathode} (mg)	M_{anode} (mg)	$M_{\text{separator}}$ (mg)	$M_{\text{electrolyte}}$ (mg)	M_{total} (mg)	Specific energy (Wh kg^{-1})
2.41	2.72	20.29	15.09	3.02	6.80	44.97	228
5.38	6.07	37.29	15.09	3.02	15.18	70.36	326
7.14	8.08	47.60	15.09	3.02	20.19	85.67	356
9.25	10.45	60.30	15.09	3.02	26.14	104.32	378
11.98	13.54	76.97	15.09	3.02	33.86	128.71	397
14.59	16.49	93.30	15.09	3.02	41.22	152.41	409
16.24	18.35	101.41	15.09	3.02	45.88	165.17	420
17.99	20.33	111.30	15.09	3.02	50.82	180.00	427
20.18	22.70	124.50	15.09	3.02	56.75	199.13	431

The energy density of the cell is calculated according to,

(4) $$\text{Energy density (Wh L}^{-1}\text{)} = \frac{\text{Energy}}{\text{Volume of the cell}} = \frac{\text{Nominal voltage} \times C/A}{T_{cathode} + T_{anode} + T_{separator}}$$

where T_{cathode}, T_{anode}, $T_{\text{separator}}$ are the thickness of cathode (including 16 μm of Al foil), anode (including 9 μm of Cu foil) and separator, respectively.

C/*A* (mAh cm^{-2})	T_{cathode} (μm)	T_{anode} (μm)	$T_{\text{separator}}$ (μm)	Energy density (Wh L^{-1})
2.41	61	109	16	490
5.38	114	109	16	852
7.14	147	109	16	993
9.25	187	109	16	1122
11.98	240	109	16	1242
14.59	291	109	16	1327
16.24	318	109	16	1386
17.99	348	109	16	1438
20.18	390	109	16	1482

Supplementary Table 2 | Comparison of the *C/A*, C_{sp}, ratio of C_{sp}/theoretical C_{sp}, and specific energy of the electrode reported in this study and previously reported high-*C/A* electrodes (mostly based on coin-type cells). For fair comparison, the specific energies were estimated without including the packaging substances because most previous studies did not provide information on the packaging substances.

Reference	Type of cell	*C/A* (mAh cm^{-2})	C_{sp} (mAh g^{-1})	Utilization (C_{sp}/theoretical C_{sp} = %)	Specific energy (Wh kg^{-1})
This study	Double-stacked pouch	20	210 (NCM811)	100	431
1	Mono-stacked pouch	9	112 (NCM111)	72	206
2	Mono-stacked pouch	6	158 (NCM622)	93	246
3	Mono-stacked pouch	12	203 (NCM811)	97	404
4	Coin	8	128 (LCO)	88	-
5	Coin	21	144 (LFP)	85	-
6	Coin	2	150 (LFP)	88	-
7	Coin	14	133 (LFP)	78	-
8	Coin	7	140 (LFP)	82	-
9	Coin	9	130 (LFMP)	86	-
10	Coin	14	142 (LCO)	97	-
11	Coin	22	106 (LFP)	73	219
12	Coin	7	126 (LFP)	74	-
12	Coin	23	158 (LFP)	93	-
13	Coin	28	132 (LCO)	91	-

14	Coin	10	112<br>(NCM111)	72	205
15	Coin	29	185<br>(NCM811)	95	401
16	Coin	13	162<br>(OLO)	65	-
17	Coin	16	152<br>(LFP)	89	-
18	Coin	9	146<br>(LFP)	85	-

Supplementary Table 3 | Details on the calculation of the specific energy and energy density of the double-stacked pouch-type full cell (including the packaging substance). The area of the cathode and anode in the cell is 34 × 34 mm^2 and 36 × 36 mm^2, respectively.

Cell components	Mass (mg)	*M*/*A* ($mg\ cm^{-2}$)	*C*/*A* ($mAh\ cm^{-2}$)	Thickness (μm)
Cathode #1	1143	98.87	18.05	348
Cathode #2	1139	98.52	17.99	347
Anode	266	20.55	20 (per single side)	209
Separator	17	-	-	32
Electrolyte	1042	-	-	-
Pouch packaging	230	-	-	117

Pouch parameter	*Stack	Total
Mass (mg)	3607	4191
Thickness (μm)	936	1166
Capacity (mAh)	416	
Energy (mWh)	1554	
Specific energy ($Wh\ kg^{-1}$)	437	376
Energy density ($Wh\ L^{-1}$)	1457	1043

*Stack represents core of the pouch cell excluding pouch packaging substances.

Supplementary Table 4 | Details of the calculation of the D_{Li+} of the c-IPN (vs. n-IPN) cathodes.

The D_{Li+} was calculated using the following equation (2)[13]:

$$D_{Li^+} = \frac{4}{\pi \Delta t}\left(\frac{m_B V_M}{M_B S}\right)\left(\frac{\Delta E_s}{\Delta E_t}\right)^2 \quad (2)$$

where m_B is assigned to the mass of the electrode active material, S is the geometric area of the electrode, M_B is the molar mass of the electrode material, V_M is the molar volume of the electrode material, and other parameters (Δt, ΔE_t, and ΔE_s) in the equation were displayed in the **Supplementary Fig. 19b**.

	Δt (s)	M_B (g mol^{-1})	V_M (cm^3 mol^{-1})	$m_B S^{-1}$ (mg cm^{-2})	ΔE_s (mV)	ΔE_t (mV)	D_{Li+} (cm^2 s^{-1})
n-IPN	3600	97.28	20.53	64.56	61.11	158.98	9.69 E-09
c-IPN	3600	97.28	20.53	64.48	51.54	86.52	2.32 E-08

Supplementary Table 5 | Analysis of the equivalent circuit elements (obtained from the Nyquist plots in **Supplementary Fig. 22**) as a function of cycle number.

Cathode	Impedance (ohm cm^2)	1st cycle	50th cycle
n-IPN	R_{film}	9.3	79.8
	R_{ct}	17.0	47.0
c-IPN	R_{film}	9.2	23.6
	R_{ct}	4.0	33.6

Supplementary Table 6 | Detailed information of the model systems of the n-IPN and c-IPN binder. The initial densities of the systems were obtained from the experimental results. Initial and final configurations for MD simulations are included in **Supplementary Data 1–4**.

		Bulk n-IPN	Bulk c-IPN	n-IPN/electrolyte	c-IPN/electrolyte
Number of	TMTPA chains	17	7	17	7
	VAI-TFSI chains	-	10	-	10
	EC	-	-	560	650
	DEC	-	-	308	358
	$LiPF_6$	-	-	143	166
Size of the system (nm^3)		4.1×4.1×4.1	4.4×4.4×4.4	4.1×4.1×14.0	4.4×4.4×14.5

Supplementary Reference

1 Yu, Z. *et al.* Molecular design for electrolyte solvents enabling energy-dense and long-cycling lithium metal batteries. *Nat. Energy* **5**, 526-533 (2020).

2 Lee, Y.-G. *et al.* High-energy long-cycling all-solid-state lithium metal batteries enabled by silver–carbon composite anodes. *Nat. Energy* **5**, 299-308 (2020).

3 Louli, A. J. *et al.* Diagnosing and correcting anode-free cell failure via electrolyte and morphological analysis. *Nat. Energy* **5**, 693-702 (2020).

4 Qiao, Y. *et al.* A high-energy-density and long-life initial-anode-free lithium battery enabled by a Li_2O sacrificial agent. *Nat. Energy* **6**, 653-662 (2021).

5 Ren, X. *et al.* Enabling High-Voltage Lithium-Metal Batteries under Practical Conditions. *Joule* **3**, 1662-1676 (2019).

6 Lin, L. *et al.* Li-Rich Li2 $[Ni_{0.8}Co_{0.1}Mn_{0.1}]O_2$ for Anode-Free Lithium Metal Batteries. *Angew Chem. Int. Ed. Engl.* **60**, 8289-8296 (2021).

7 Kim, J. H., Kim, J. M., Cho, S. K., Kim, N. Y. & Lee, S. Y. Redox-homogeneous, gel electrolyte-embedded high-mass-loading cathodes for high-energy lithium metal batteries. *Nat. Commun.* **13**, 2541 (2022).

8 Park, K.-Y. *et al.* Understanding capacity fading mechanism of thick electrodes for lithium-ion rechargeable batteries. *J. Power Sources* **468**, 228369 (2020).

9 Kim, H. *et al.* Failure mode of thick cathodes for Li-ion batteries: Variation of state-of-charge along the electrode thickness direction. *Electrochim. Acta* **370**, 137743 (2021).

10 Keeler, J. Understanding NMR spectroscopy, 2nd ed. *Wiley: Chichester* (2010).

11 Ogihara, N. *et al.* Theoretical and Experimental Analysis of Porous Electrodes for Lithium-Ion Batteries by Electrochemical Impedance Spectroscopy Using a Symmetric Cell. *J. Electrochem. Soc.* **159**, A1034-A1039 (2012).

12 H. Sun, L. M. Three-dimensional holey-graphene/niobia composite architectures for ultrahigh-rate energy storage. *Science* **356**, 599 (2017).

13 Weppner, W. & Huggins, R. A. Determination of the kinetic parameters of mixed-conducting electrodes and application to the system Li_3Sb. *J. Electrochem. Soc.* **124**, 1569 (1977).

14 Thomas, M., Bruce, P. G. & Goodenough, J. B. AC impedance analysis of polycrystalline insertion electrodes: application to $Li_{1-x}CoO_2$. *J. Electrochem. Soc.* **132**, 1521 (1985).

15 Fan, X. *et al.* Non-flammable electrolyte enables Li-metal batteries with aggressive cathode chemistries. *Nat. Nanotechnol.* **13**, 715-722 (2018).

16 Liu, W. *et al.* Inhibition of transition metals dissolution in cobalt-free cathode with ultrathin

robust interphase in concentrated electrolyte. *Nat. Commun.* **11**, 3629 (2020).
17 Fan, X. *et al.* Crack-free single-crystalline Ni-rich layered NCM cathode enable superior cycling performance of lithium-ion batteries. *Nano Energy*, **70**, 104450 (2020).
18 Yan, P. *et al.* Tailoring grain boundary structures and chemistry of Ni-rich layered cathodes for enhanced cycle stability of lithium-ion batteries. *Nat. Energy* **3**, 600-605 (2018).
19 Xu, G.-L. *et al.* Building ultraconformal protective layers on both secondary and primary particles of layered lithium transition metal oxide cathodes. *Nat. Energy* **4**, 484-494 (2019).
20 Park, S.-H. *et al.* High areal capacity battery electrodes enabled by segregated nanotube networks. *Nat. Energy* **4**, 560-567 (2019).
