# Supplementary material for: Regulating electrostatic phenomena by cationic polymer binder for scalable high-areal-capacity Li battery electrodes
Source: Nat Commun. 2023 Sep 15;14:5721. doi: 10.1038/s41467-023-41513-1 (PMC10504278; doi:10.1038/s41467-023-41513-1)
Supplement: Supplementary file 3 — Description of additional supplementary files [file 41467_2023_41513_MOESM3_ESM.pdf]

<u>Description of Additional Supplementary Files Document</u>

Supplementary Data 1

The initial configuration of n-IPN/Electrolyte system, converted to mol2 format

Supplementary Data 2

The final configuration of n-IPN/Electrolyte system, converted to mol2 format

Supplementary Data 3

The initial configuration of c-IPN/Electrolyte system, converted to mol2 format

Supplementary Data 4

The final configuration of c-IPN/Electrolyte system, converted to mol2 format
